# Genistein Suppresses Prostate Cancer Growth through Inhibition of Oncogenic MicroRNA-151

**DOI:** 10.1371/journal.pone.0043812

**Published:** 2012-08-23

**Authors:** Takeshi Chiyomaru, Soichiro Yamamura, Mohd Saif Zaman, Shahana Majid, Guoren Deng, Varahram Shahryari, Sharanjot Saini, Hiroshi Hirata, Koji Ueno, Inik Chang, Yuichiro Tanaka, Z. Laura Tabatabai, Hideki Enokida, Masayuki Nakagawa, Rajvir Dahiya

**Affiliations:** 1 Department of Urology, San Francisco Veterans Affairs Medical Center and University of California San Francisco, San Francisco, California, United States of America; 2 Department of Pathology, San Francisco Veterans Affairs Medical Center and University of California San Francisco, San Francisco, California, United States of America; 3 Department of Urology, Graduate School of Medical and Dental Sciences, Kagoshima University, Kagoshima, Japan; Wayne State University School of Medicine, United States of America

## Abstract

Genistein has been shown to suppress the growth of several cancers through modulation of various pathways. However, the effects of genistein on the regulation of oncogenic microRNA-151 (miR-151) have not been reported. In this study, we investigated whether genistein could alter the expression of oncogenic miR-151 and its target genes that are involved in the progression and metastasis of prostate cancer (PCa). Real-time RT-PCR showed that the expression of miR-151 was higher in PC3 and DU145 cells compared with RWPE-1 cells. Treatment of PC3 and DU145 cells with 25 µM genistein down-regulated the expression of miR-151 compared with vehicle control. Inhibition of miR-151 in PCa cells by genistein significantly inhibited cell migration and invasion. *In-silico* analysis showed that several genes (CASZ1, IL1RAPL1, SOX17, N4BP1 and ARHGDIA) suggested to have tumor suppressive functions were target genes of miR-151. Luciferase reporter assays indicated that miR-151 directly binds to specific sites on the 3′UTR of target genes. Quantitative real-time PCR analysis showed that the mRNA expression levels of the five target genes in PC3 and DU145 were markedly changed with miR-151 mimics and inhibitor. Kaplan-Meier curves and log-rank tests revealed that high expression levels of miR-151 had an adverse effect on survival rate. This study suggests that genistein mediated suppression of oncogenic miRNAs can be an important dietary therapeutic strategy for the treatment of PCa.

## Introduction

Prostate cancer (PCa) is one of the most common malignancies among men and ranks second to lung cancer in cancer-related deaths [1]. After androgen-deprivation therapy. PCa may most recur as androgen-independent, metastatic disease that leads to death within several years [2]. Currently, no effective therapies are available to cure androgen-independent PCa. Thus, new prognostic markers and effective treatment strategies are urgently needed.

MicroRNAs (miRNAs) are a class of small non-coding RNA of approximately 22 nucleotides that regulate gene expression through translational repression and mRNA cleavage [3]. Bioinformatics indicate that miRNAs regulate 60% of protein-coding genes [4]. At present, 1,527 human miRNAs have been registered in the miRBase database (http://microrna.sanger.ac.uk/). miRNAs are involved in a variety of biological processes, including metabolism, development, and differentiation, and contribute to the development of various types of cancer [5]. Many human cancers have aberrant expression of miRNAs, which can function either as tumor suppressors or oncogenes [6].

**Figure 1:**
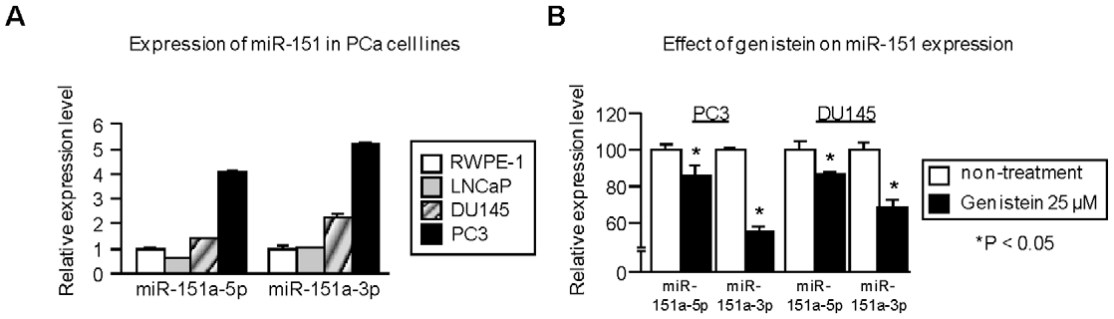
Expression of miR-151 in PCa cells and effect of genistein treatment. (A) Expression profile of miR-151 in PCa cell lines (LNCaP, DU145 and PC3) and normal prostate epithelial cells (RWPE-1). Real-time PCR showed that the expression levels of miR-151 were up-regulated in androgen-independent PCa cell lines (DU145 and PC3). miR-151 expression was normalized to RNU48. Data are presented as means ± SE. (B) Expression levels of miR-151 after treatment with genistein (25 µM). miR-151 expression was found to be reduced by 15–45% in genistein treated cells. *,P<0.05.

miR-151 is mapped to a region of chromosome 8q. That has been found to be frequently amplified in several cancers including bladder, kidney, prostate, breast, lung, gastric and rectal cancer [7]–[13]. We previously demonstrated that chromosomal gain of locus 8q24.3, where oncogenic LY6K gene resides, may have a critical role in bladder cancer development [7]. One paper showed that copy number gain of the miR-151 gene at 8q24.3 in PCa was correlated with metastasis [14].

Genistein (4′,5,7-Trihydroxyisoflavone), a major isoflavone constituent of soybeans and soy products, has been shown to exhibit potent anticancer effects on PCa [15], [16]. Epidemiological evidence indicate that the incidence and mortality rates of PCa are considerably lower in Asia compared to the United States [17]. The mean serum concentration of genistein in Asian men was higher than that of the US population [18] and several studies have demonstrated that isoflavone intake was associated with a reduction in PCa risk [19]–[22]. Genistein has multiple molecular targets including receptors, enzymes, and signaling pathways [15]. Genistein has also been shown to suppress the growth of several cancer cell lines *in vitro* and *in vivo*, reducing the expression of several oncogenic miRNAs. To date, the effects of genistein on the regulation of miR-151 have not been reported. Therefore in this study we investigated whether genistein could alter the expression of miR-151 and its target genes involved in the progression and metastasis of PCa.

**Figure 2:**
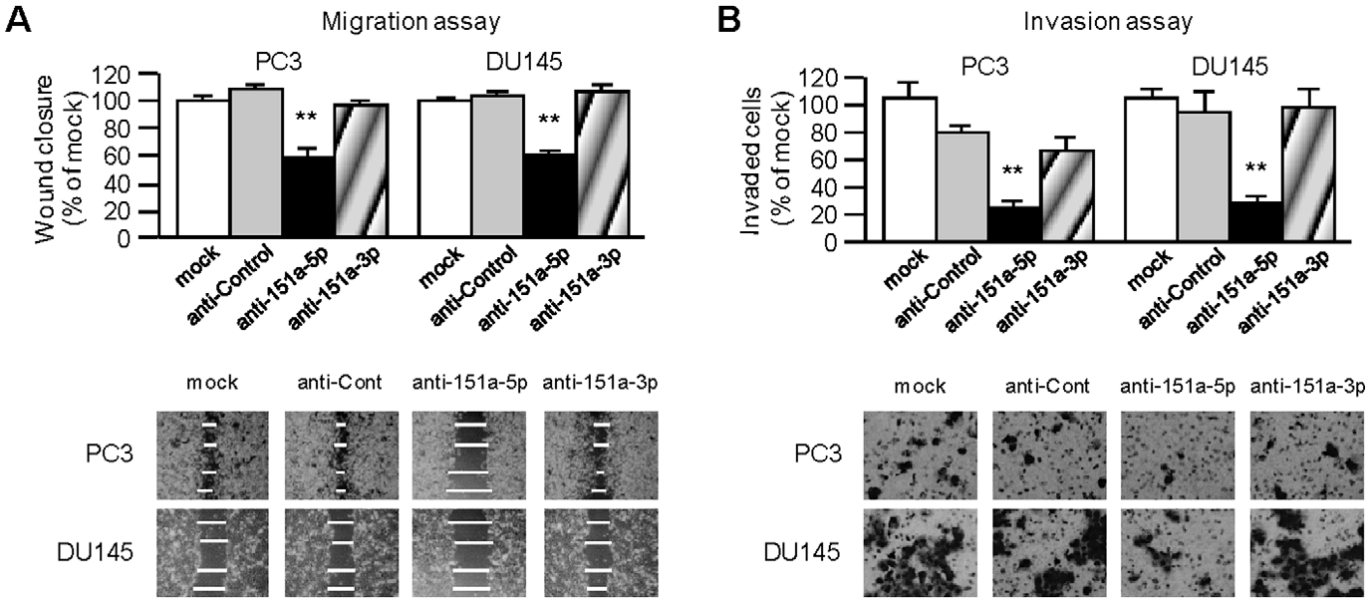
Effect of miR-151a-5p knockdown on prostate cancer cell migration and invasion. (A) Knockdown of miR-151a-5p significantly inhibits cell migration. After transfection (48 hours), a wound was formed by scraping and the wound measured after 24 hours. Representative images of wound healing assay are shown at x200 magnification. **,P<0.0001 (B) Knockdown of miR-151a-5p significantly decreased cell invasion. Representative images of invasion assay are shown at x200 magnification. **,P<0.0001.

## Results

### miR-151 is Up-regulated in PCa and Genistein Treatment Decreased miR-151

To determine relative expression levels of miR-151 in PCa cells, we performed TaqMan quantitative real-time PCR analysis using androgen-dependent (LNCaP) and androgen-independent (PC3, DU145) cell lines and compared these with normal prostate epithelial cells (RWPE-1). miR-151 consists of two mature miRNAs; miR-151a-5p and miR-151a-3p (miRBase). We observed that miR-151 expression was markedly up-regulated in androgen-independent PCa cell lines compared to RWPE-1 cells (miR-151a-5p; PC3 4.02-fold, DU145 1.36-fold) (miR-151a-3p; PC3 5.14-fold, DU145 2.25-fold) (Fig. 1A).

Genistein treatment significantly down-regulated the relative expression level of miR-151 compared with vehicle control (miR-151a-5p; PC3 15% decrease, DU145 14% decrease) (miR-151a-3p; PC3 44% decrease, DU145 32% decrease) (Fig. 1B).

### Effect of miR-151 Knockdown on Cell Proliferation, Migration, and Invasion in PCa Cell Lines

To examine the functional roles of miR-151, we performed loss-of-function studies using anti-miR miRNA inhibitor transfected into PC3 and DU145 cells. The expression of miR-151 was markedly repressed in anti-miR miRNA inhibitor transfectants (data not shown) and we observed similar cell viability in all cell lines, suggesting that knockdown miR-151 did not affect cell proliferation (data not shown). Wound healing assay demonstrated significant inhibition of cell migration in anti-miR miRNA-151a-5p transfected PCa cell lines compared with their counterparts (Fig. 2A). However, no significant inhibition was observed with anti-miR miRNA-151a-3p transfected PCa cell lines. The Matrigel invasion assay demonstrated that the number of invading cells was significantly decreased in anti-miR miRNA-151a-5p transfectants compared with their counterparts (Fig. 2B). Therefore it is likely that miR-151a-5p plays on important role in tumor cell migration and invasion. To evaluate the simultaneous effects of miR-151a-5p and miR-151a-3p, loss-of-function studies using miRNA inhibitor co-transfected PCa cell lines was carried out. There was no additional effect on cell viability by miR-151a-5p and miR-151a-3p co-transfection (data not shown).

**Figure 3:**
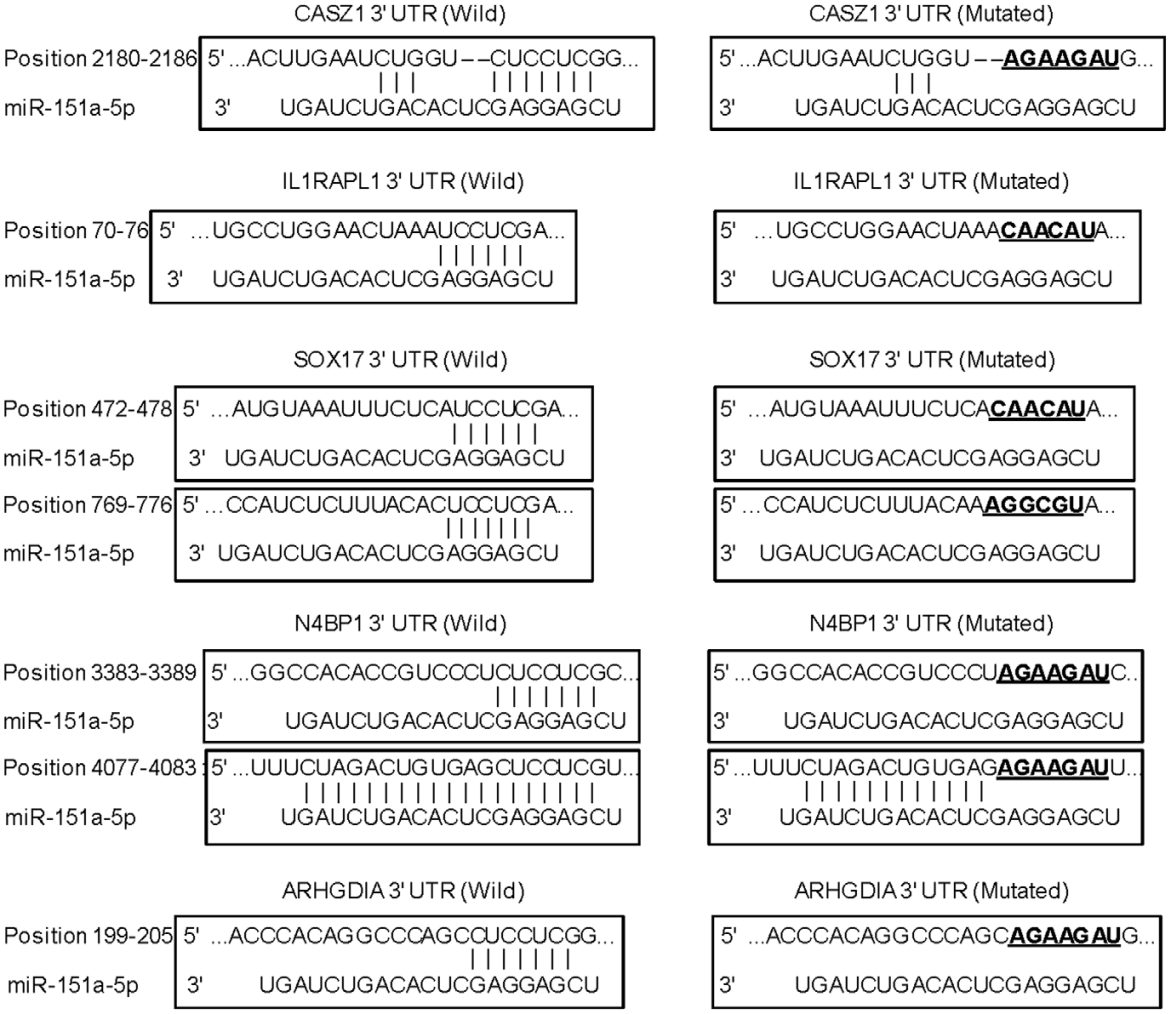
Putative miR-151a-5p binding and mutated sites in the 3′UTR of target genes.

**Figure 4:**
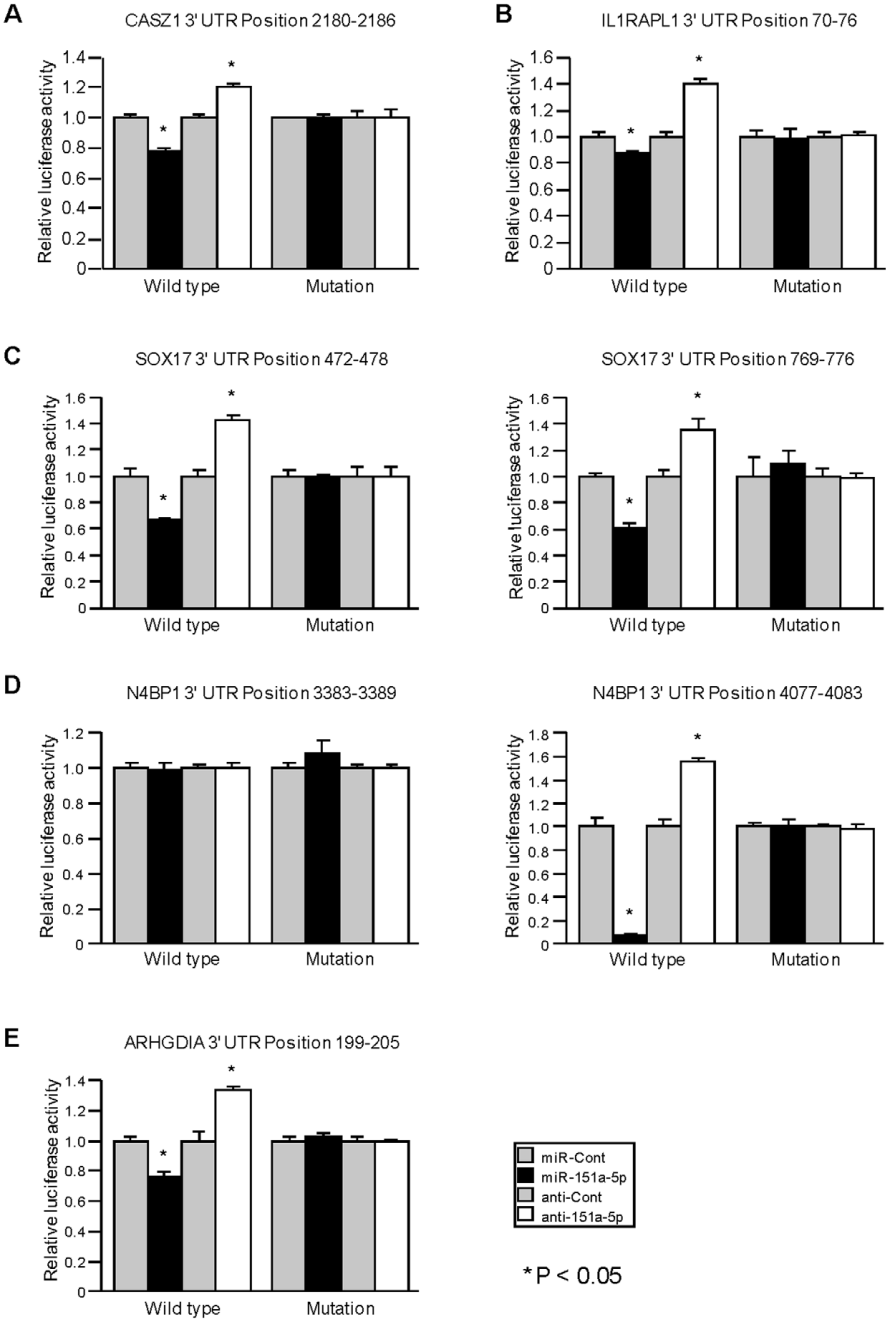
Luciferase reporter assays using the vectors encoding putative target sites of 3′UTR. PC-3 cells were transiently transfected with Pre-miR miRNA precursor or Anti-miR miRNA Inhibitor or negative control, followed by transient transfection with wild-type 3′UTR reporter plasmids or mutated 3′UTR plasmids for 24 hours. 3′UTR reporter activity was measured by luciferase assay and normalized to activity of Renilla luciferase. Data are presented as the mean ± SE. *,P<0.05.

### Identification of miR-151a-5p Target Genes by In-silico Analysis

To identify potential target genes of miR-151a-5p, we used TargetScan and microRNA.org to identify sites where these miRNAs bind. These programs identified five genes containing putative target sites for miR-151a-5p in their 3′UTR (N4BP1: NEDD4 binding protein 1, CASZ1: castor zinc finger 1, IL1RAPL1: interleukin 1 receptor accessory protein-like 1, SOX17: SRY (sex determining region Y)-box 17 and ARHGDIA: Rho GDP dissociation inhibitor (GDI) alpha. These genes were identified by both algorithms and bioinformatic analysis showed they may have tumor suppressor function in several cancers [23]–[27].

**Figure 5:**
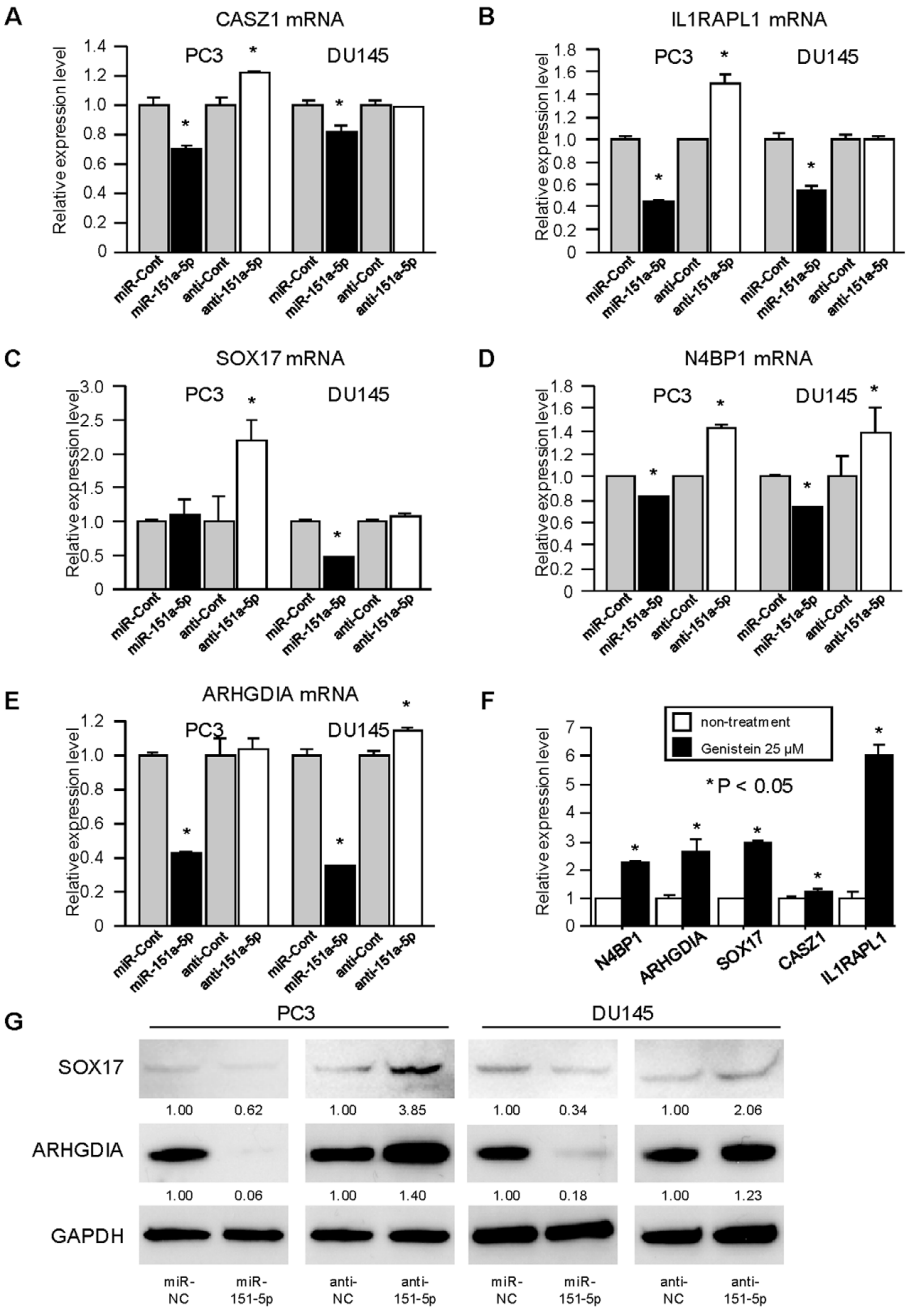
miR-151a-5p targets CASZ1, IL1RAPL1, SOX17, N4BP1 and ARHGDIA genes. (A) (B) (C) (D) (E) The mRNA levels of five target genes of miR-151a-5p were determined by quantitative real-time PCR analyses after transfection with miR-151a-5p mimics, miR-151a-5p inhibitor and negative control in PCa cell lines (PC3 and DU145). (F) The mRNA levels of five target genes of miR-151a-5p were determined by quantitative real-time PCR analyses after treatment with genistein (25 µM) in PCa cell lines (DU145). (G) The protein levels of target genes of miR-151a-5p were determined by western blot analyses after transfection with miR-151a-5p mimics, miR-151a-5p inhibitor and negative control in PCa cell lines (PC3 and DU145). Data are presented as the mean ± SE. *,P<0.05.

### Luciferase Reporter Assays Using Vectors Containing 3′UTR Binding Sites of Putative Target Genes

CASZ1, IL1RAPL1 and ARHGDIA each have one predicted binding site for miR-151a-5p in their 3′UTRs while N4BP1 and SOX17 each have two predicted binding sites (Fig. 3). We cloned the putative miR-151a-5p 3′UTRs targets into a luciferase reporter assay vector. Luciferase reporter assay demonstrated that miR-151a-5p decreased the relative luciferase activities of CASZ1, IL1RAPL1 and ARHGDIA (Fig. 4A, 4B and 4E). Luciferase reporter assay also demonstrated that suppression of miR-151a-5p increased the relative luciferase 3′UTR activities. Mutation of the putative miR-151a-5p binding sites in these 3′UTRs decreased the response to miR-151a-5p. These results indicate that miR-151a-5p binds directly to the 3′UTRs of CASZ1, IL1RAPL1 and ARHGDIA. Luciferase reporter assay also demonstrated that miR-151a-5p decreased the relative luciferase activities, and knockdown of miR-151a-5p increased the relative luciferase activities with both wild-type SOX17 3′UTRs (Position 472–478 and Position 769–776) (Fig. 4C and 4D). N4BP1 3′UTR Position 3383–3389 showed no luciferase activity change with either miR-151a-5p mimics or miR-151a-5p inhibitor. The other target site (Position 4077–4083) showed that miR-151a-5p markedly decreased the relative luciferase activity and suppression of miR-151a-5p significantly increased luciferase activity with the wild-type 3′UTR. Thus these data suggest that miR-151a-5p directly binds to one site (Position 4077–4083) on the 3′UTR of N4BP1 mRNA.

**Figure 6:**
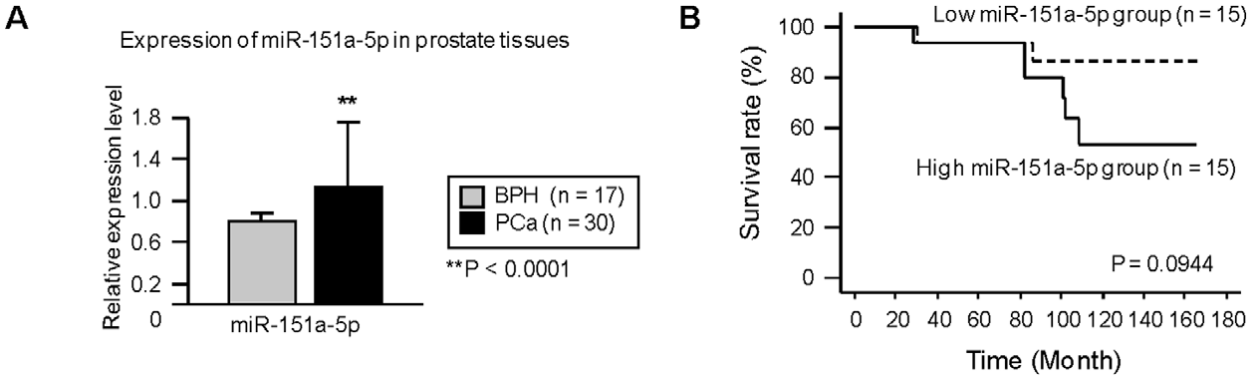
Expression of miR-151-5p in PCa specimens. (A) miR-151 expression in clinical samples (BPH, n = 17; PCa, n = 30). **,P<0.0001 (B) Kaplan-Meier survival curve showing overall survival by miR-151a-5p expression (P = 0.0944).

### Regulation of Target Gene Expression in PCa Cell Lines by miR-151a-5p

Quantitative real-time PCR analysis showed that the mRNA expression levels of the five target genes in PC3 and DU145 were markedly repressed in the miR-151a-5p transfectants compared with the controls (Fig. 5A–E). In addition, mRNA expression of the five target genes in PC3 and DU145 were markedly up-regulated in the miR-151a-5p inhibitor transfectants compared with the controls. We also evaluated the effects of genistein on the mRNA expression of miR-151a-5p targets. These target genes were almost up-regulated with genistein treatment in DU145 cell lines (Fig. 5F). The protein expression levels of SOX17 and ARHGDIA were significantly decreased in the miR-151a-5p transfectants compared with the controls and up-regulated in the miR-151a-5p inhibitor transfectants (Fig. 5G).

### Up-regulated Expression of miR-151-5p in PCa Specimens

We evaluated expression levels of miR-151-5p in PCa (n = 30) and benign prostate cancer (BPH) (n = 17) tissues. The expression levels of miR-151-5p were significantly higher in PCa compared with BPH tissues (P = 0.0001; Fig. 6A). To determine if the levels of miR-151a-5p in tumor tissues correlates with survival of the PCa patients, we also measured miR-151a-5p expression levels in human tumor samples (Fig. 6B). The results show that patients with high miR-151a-5p expression had a lower survival rate than those with low 151a-5p expression, though these results did not reach statistical significance (P = 0.0944). There was also no significant correlation with another clinico-pathological factors (tumor stage and Gleason Score) (data not shown).

**Table 1:** Prostate cancer patient information.

Characteristic		(%)	
Age (years)			
Median (range)	62 (50–78)		
PSA (ng/ml)			
Median (range)	5.05 (0.2–298.9)		
Total number	30	(100.0)	
Gleason Score			
GS 6	17	(56.7)	
GS 7	10	(33.3)	
GS 8	2	(6.7)	
GS 9	1	(3.3)	
Pathological tumor stage			
pT2a	3	(10.0)	
pT2b	6	(20.0)	
pT2c	11	(36.7)	
pT3a	4	(13.3)	
pT3b	1	(3.3)	
unknown	5	(16.7)	

Abbreviations: PSA = prostate-specific antigen; GS = Gleason Score.

## Discussion

In this study we have demonstrated that the miR-151 is highly expressed in androgen-independent PCa cell lines and that cell motility and invasiveness are reduced in miR-151a-5p knockdown cell lines (PC3 and DU145). miR-151 consists of two mature miRNAs; miR-151a-5p and miR-151a-3p. Two mature miRNAs are excised from the same stem-loop precursor-miR-151a. Thus miR-151a-5p and miR-151a-3p have different sequences and therefore target different mRNAs. Wound healing assay and invasion assay demonstrated that knockdown of miR-151a-5p, but not miR-151a-3p, significantly decreased PCa cell migration and invasion. These results suggest that miR-151a-5p functions as an oncogene by increasing cell migration and invasion in PCa. In contrast, there was no significant effect on cell proliferation in miR-151a-5p inhibitor transfected PCa cells, suggesting that miR-151a-5p is not involved in proliferation activity. In this study, we showed that up-regulation of miR-151a-5p expression in tumors is related to lower survival of PCa patients however these results were not significantly different with either this parameter or other clinic-pathological features. Since our cohort was not large (n = 30) and included only five samples of advanced cancer (more than pT3) and three samples with a Gleason Score of 8 or more, studies on a larger number of samples with a balanced pathological background will have to be conducted for more precise statistical evaluation.

Computational bioinformatics and 3′ luciferase reporter assays indicate that miR-151a-5p has several potential target genes (CASZ1, IL1RAPL1, SOX17, N4BP1 and ARHGDIA). CASZ1, a neuronal differentiation gene, have been shown to possess tumor suppressor activity. In clinical primary tumor sumples, the expression of CASZ1 is significantly decreased in aggressive neuroblastoma compared with the favorable tumors [28]. The restoration of CASZ1 expression inhibits tumor migration *in vitro* and suppressed tumorigenicity *in vivo* [23]. The IL1RAPL1 gene is located at a region on chromosome X and the protein encoded by this gene is a member of the interleukin 1 receptor family and is similar to interleukin 1 accessory proteins [29]. IL1RAPL1 has been reported to be downregulated in many brain tumor cell lines and xenografts compared with normal tissues [24] suggesting that it may function as a tumor suppressor [30]. The SOX17 gene encodes a member of the SOX family of transcription factors involved in the regulation of embryonic development and in the determination of the cell fate [31]. Real time quantitative PCR showed that SOX17 expression was lower in gastric cancer tissues than those in normal tissues [32]. SOX17 was reported to be a candidate tumor suppressor gene that inhibits the canonical WNT/β-catenin signaling pathway in colorectal cancer and hepatocellular carcinoma [25], [33]. N4BP1 has been identified as a substrate for mono ubiquitylation by E3 ubiquitin ligase Nedd4 [34]. It has been shown that N4BP1 interacts with and negatively regulates ITCH E3 activity directed toward its substrates including c-Jun and p53-related tumor suppressor proteins (p73 and p63) [35]. ARHGDIA is a cellular regulatory protein that binds to and negatively regulates most Rho GTPases including RhoA, Rac1, and Cdc42 [36]. Loss of ARHGDIA enhances metastasis and resistance to tamoxifen in breast cancer [37] and loss of ARHGDIA expression promotes the development and progression of PCa [27]. In thisb study we showed that the mRNA expression levels of these five tumor suppressor target genes in PC3 and DU145 were markedly changed in experimentation with miR-151a-5p mimics and miR-151a-5p inhibitor, indicating that miR-151-5p directly targets these genes.

Genistein has been shown to suppress the growth of several cancer cell lines *in vitro* and *in vivo*, reducing the expression of oncogenic miRNAs, such as miR-21 [38], miR-27a [39], miR-221 and miR-222 [40]. In this study, we showed that genistein treatment significantly down-regulated the relative expression level of oncogenic miR-151. Recently, our group showed that genistein inhibited the expression of miR-21 in kidney cancer cells and in the tumors formed after injecting genistein treated kidney cancer cells in nude mice along with inhibition of tumor formation [38]. miR-27a has been reported to be a oncogenic miRNA in various cancer cells, and its expression and target gene (ZBTB10) levels were dependent on the dose of genistein [39]. We have previously demonstrated that genistein upregulated tumor suppressor gene ARHI by downregulating miR-221 and miR-222 in PCa [40]. Genistein also has been reported to suppress the growth of several cancers by increasing the expression of the tumor suppressors, miR-146a [41] and miR-1296 [42]. Treatment of pancreatic cancer cells with isoflavone compounds (including 70.54% genistein), increased miR-146a expression, causing downregulation of EGFR, MTA-2, IRAK-1, and NF-κB, resulted in inhibition of cell invasion [41]. We have also reported that genistein increased miR-1296 expression (3 to 5-fold) in PCa cells and significantly downregulated the expression of MCM2 which is target of miR-1296 [42].

In this study, we have shown that miR-151 directly targets several tumor suppressor genes and involved in the progression and metastasis of PCa. In addition, this is the first report to show that genistein downregulates miR-151 expression suggesting that genistein may serve as an important dietary therapeutic agent for the treatment of PCa.

## Materials and Methods

### Clinical Prostate Specimens

All tissue slides were reviewed by a board certified pathologist for the identification of prostate cancer foci as well as adjacent normal glandular epithelium. All cancer patients had elevated levels of prostate specific antigen (PSA) and had undergone radical prostatectomy from 1999 to 2004. The patients’ characteristics are shown in Table 1. Non-cancerous tissues were obtained from BPH patients who had undergone prostatectomy or transurethral resection. Informed consent was obtained from all patients.

### Cell Culture

Human prostate cancer cell lines, LNCaP, PC-3 and DU145 and a non-malignant epithelial prostate cell line, RWPE-1, were purchased from The American Type Culture Collection (Manassas, VA, USA). The prostate cancer cell lines were cultured in RPMI 1640 medium supplemented with 10% fetal bovine serum (FBS) in a humidified atmosphere of 5% CO2 and 95% air at 37°C. RWPE-1 cell line was cultured in keratinocyte growth medium supplemented with 5 ng/mL human recombinant epidermal growth factor and 0.05 mg/mL bovine pituitary extract (Invitrogen, Carlsbad, CA, USA). Subconfluent cells (60%-70% confluent) were treated with genistein (25 µmol/L; Sigma, St Louis, MO, USA) dissolved in dimethylsulfoxide and cells treated with vehicle (dimethylsulfoxide) served as control. Cell media and genistein were changed every day and grown for 4 days.

### RNA Extraction

RNA was extracted from FFPE human samples using a miRNeasy formalin-fixed paraffin-embedded kit (Qiagen, Valencia, CA, USA) after microdissection. To digest DNA, the Qiagen RNase-Free DNase kit was used. Total RNA was also extracted from prostate cancer cell lines and a non-malignant epithelial prostate cell line using a miRNeasy mini kit (Qiagen) according to the manufacturer’s instructions.

### Quantitative Real-time PCR

Extracted total RNA was reverse transcribed into single-stranded cDNA using an iScript cDNA Synthesis Kit (Bio-Rad, Hercules, CA, USA) and a TaqMan MicroRNA Reverse Transcription Kit (Applied Biosystems, Foster City, CA, USA). Quantitative real-time PCR analysis was performed with an Applied Biosystems Prism7500 Fast Sequence Detection System using TaqMan universal PCR master mix according to the manufacturer’s protocol (Applied Biosystems). Levels of RNA expression were determined using the 7500 Fast System SDS software version 1.3.1 (Applied Biosystems). Quantitative PCR parameters for cycling were as follows: 95°C for 20 seconds, 40 cycles of PCR at 95°C for 3 seconds, and 60°C for 30 seconds. All reactions were done in a 10-µL reaction volume in triplicate. The data were analyzed with the delta-delta Ct method to calculate the fold-change. TaqMan probes and primers for CASZ1 (assay ID: Hs00214901_m1), IL1RAPL1 (assay ID: Hs00990788_m1), SOX17 (assay ID: Hs00751752_s1), N4BP1 (assay ID: Hs00206373_m1), ARHGDIA (assay ID: Hs00366348), GAPDH (assay ID: Hs02758991_g1), miR-151a-5p (assay ID: 002642), miR-151a-3p (assay ID: 002254), RNU48 (Assay ID: 001006) were obtained from Applied Biosystems. GAPDH and RNU48 were used as internal controls.

### Western Blot Analysis

At 72 hours after transfection, cells were lysed with RIPA buffer (Pierce, Brebieres, France) containing protease inhibitors (Sigma). Protein quantification was done using a BCA protein assay kit (Pierce). Protein lysate (30 µg) was separated by 4% to 20% SDS polyacrylamide gels and transferred to nitrocellulose membrane. An antibody against SOX17 was purchased from Millipore (Billerica, MA, USA). Antibodies against ARHGDIA and GAPDH were purchased from GeneTex (Irvine, CA, USA). The membrane was washed and then incubated with secondary antibodies conjugated to horseradish peroxidase (Cell Signaling Technology, Danvers, MA, USA). Specific complexes were visualized with an echochemiluminescence (ECL) detection system (GE Healthcare, Little Chalfont, UK), and the expression level of these genes was evaluated by using ImageJ software (ver. 1.43; http://rsbweb.nih.gov/ij/index.html).

### Transfection

Pre-miR miRNA precursor and negative control (Applied Biosystems) were used in the gain-of-function experiments, whereas Anti-miR miRNA Inhibitor and negative-control (Applied Biosystems) were used in the loss-of-function experiments. PC3 and DU145 cells were transiently transfected using Lipofectamine 2000 transfection reagent (Invitrogen), according to the manufacturer’s recommendations. Mock transfections, with the transfection reagent, were used as controls.

### Cell Proliferation, Migration, and Invasion Assays

Cell proliferation was measured using a CellTiter 96 AQueous One Solution Cell Proliferation Assay (MTS) (Promega, Madison, WI, USA) performed according to the manufacturer’s instructions. Cell proliferation was determined by absorbance measurements at 490 nm using SpectraMAX 190 (Molecular Devices Co., Sunnyvale, CA, USA). Cell migration activity was evaluated by a wound-healing assay. Cells were plated in six-well dishes, and the cell monolayers were scraped using a P-20 micropipette tip. The width of the initial gap (0 h) and the residual gap 24 hours after wounding were calculated from photomicrographs. A cell invasion assay was carried out using modified Boyden Chambers consisting of transwell-precoated Matrigel membrane filter inserts with eight micron pores in 24-well tissue culture plates (BD Biosciences, Bedford, MA, USA). Minimum essential medium containing 10% FBS in the lower chamber served as the chemoattractant, as described previously [43]. All experiments were performed in triplicate.

### Prediction of MicroRNA Targets

The predicted target genes and their miRNA binding site seed regions were determined using TargetScan (release 5.2, http://www.targetscan.org/) and microRNA.org (August 2010 release, http://www.microrna.org/microrna/home.do). The sequences of the predicted mature miRNAs were confirmed by miRBase (release 18.0; http://microrna.sanger.ac.uk/).

### Plasmid Construction and Dual-luciferase Reporter Assays

For 3′ UTR luciferase reporter assay, PmirGLO Dual-Luciferase miRNA Target Expression Vector was used (Promega). The oligonucleotide sequences (wild-type) used are shown in Table S1. We also constructed mutated oligonucleotides for each of the wild-type oligonucleotides (Table S1). In a total volume of 25 µl, 1 µl each of 100 µM forward and reverse oligonucleotide, 2.5 µl of 10 X annealing buffer (100 mM Tris–HCl, pH 7.5, 1 M NaCl and 10 mM ethylenediaminetetraacetic acid) and 20.5 µl water were incubated at 95°C for 3 min and then placed at 37°C for 15 min. The oligonucleotides were ligated into the PmeI–XbaI site of pmirGLO Dual-Luciferase miRNA Target Expression Vector. For 3′ UTR luciferase assay, prostate cancer cells were co-transfected with Pre-miR miRNA precursor or Anti-miR miRNA Inhibitor and pmirGLO Dual-Luciferase miRNA Target Expression Vectors using Lipofectamine 2000 (Invitrogen) and X-tremeGENE HP DNA Transfection Reagent (Roche Diagnosis, Basel, Switzerland, USA) according to the manufacturer’s instructions. Luciferase reporter assay was performed using the Dual-Luciferase Reporter Assay System (Promega) 24 hours after transfection.

### Statistical Analysis

The relationship between two variables and the numerical values obtained by real-time RT-PCR were analyzed using the nonparametric Mann-Whitney U test or the paired t-test. The relationship among three variables and the numerical values were analyzed using the Bonferroni-adjusted Mann-Whitney U test. Association of miR-151 expression with overall survival was estimated by the Kaplan–Meier method, and the resulting curves were compared using the log rank test. All analyses were performed using Expert StatView (version 4, SAS Institute Inc., Cary, NC, USA). In the comparison among three variables, a non-adjusted statistical level of significance of P<0.05 corresponds to a Bonferroni-adjusted level of P<0.0167.

## Supporting Information

Table S1 **Primer oligonucleotide sequences (wild-type and mutated).**
(DOC)
