# Supplementary material for: Genistein Suppresses Prostate Cancer Growth through Inhibition of Oncogenic MicroRNA-151
Source: PLoS One. 2012 Aug 23;7(8):e43812. doi: 10.1371/journal.pone.0043812 (PMC3426544; doi:10.1371/journal.pone.0043812)
Supplement: Table S1 — Primer oligonucleotide sequences (wild-type and mutated). (DOC) [file pone.0043812.s001.doc]

**Supporting Information**
